# Heterosynaptic plasticity-induced modulation of synapses

**DOI:** 10.1186/s12576-023-00893-1

**Published:** 2023-12-06

**Authors:** Masoumeh Kourosh-Arami, Alireza Komaki, Masoumeh Gholami, Seyed Hossein Marashi, Sara Hejazi

**Affiliations:** 1 Department of Neuroscience, School of Advanced Technologies in Medicine, Iran University of Medical Sciences, Tehran, Iran; 2 Department of Neuroscience, School of Science and Advanced Technologies in Medicine, Hamadan University of Medical Sciences, Hamadan, Iran; 3 Department of Physiology, Medical College, Arak University of Medical Sciences, Arak, Iran; 4 Department of Physiology, Medical College, Tarbiat Modares University, Tehran, Iran; 5 Department of Industrial Engineering & Management Systems, University of Central Florida, Orlando, USA

**Keywords:** Heterosynaptic plasticity, Homosynaptic plasticity, Synaptic weight, Dynamics

## Abstract

Plasticity is a common feature of synapses that is stated in different ways and occurs through several mechanisms. The regular action of the brain needs to be balanced in several neuronal and synaptic features, one of which is synaptic plasticity. The different homeostatic processes, including the balance between excitation/inhibition or homeostasis of synaptic weights at the single-neuron level, may obtain this. Homosynaptic Hebbian-type plasticity causes associative alterations of synapses. Both homosynaptic and heterosynaptic plasticity characterize the corresponding aspects of adjustable synapses, and both are essential for the regular action of neural systems and their plastic synapses.

In this review, we will compare homo- and heterosynaptic plasticity and the main factors affecting the direction of plastic changes. This review paper will also discuss the diverse functions of the different kinds of heterosynaptic plasticity and their properties. We argue that a complementary system of heterosynaptic plasticity demonstrates an essential cellular constituent for homeostatic modulation of synaptic weights and neuronal activity.

**Graphical Abstract:**

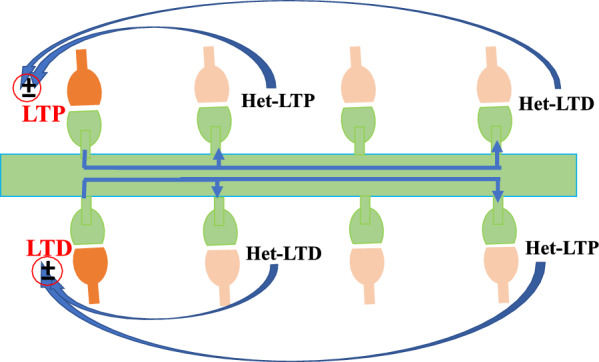

## Introduction

Synaptic weights are alterations triggered in various ways leading to various plasticity systems [1–6]. Plasticity is essential for the fundamental operations of the brain. The persistent modification of synaptic strength by neuronal activity has been considered a substrate of learning and memory. Synaptic plasticity leads to the induction and modification of connection forms in the nervous system, in addition to the adaptive behaviors responding to environmental changes throughout life. According to Hebb's proposal, if the firing of a pre-synaptic neuron is temporally associated closely with the postsynaptic neuron, the power of the association among the two neurons will also be strengthened for a long time [7]. In this strengthening of synapses—which is called "associative"—when the first neuron is activated, the possibility of the postsynaptic neuron firing is augmented [8]. Two neurons with synchronized firing indicate that a particular synapse has developed input-specific strengthening, while the rest have failed to change. Therefore, the Hebbian synapse demonstrates three features—homosynaptic plasticities, associativity, and input specificity [9, 10].

Biological neural structures have similar mechanisms, as shown via the steady action and varied dynamics of synaptic alterations in a wide range of states [11]. According to Hebb's rule [7], synaptic weights and connections frequently or persistently lead to the augmentation of postsynaptic neuronal firing. Hebbian structures of plasticity constitute the cellular representations of learning, but they also reveal an important problem relating to the stability of neural networks; the expression of long-term potentiation (LTP) may yield a damaging positive-feedback cycle and runaway excitation, while long-term depression (LTD) can result in excessive depression. Synapses that do not consistently cooperate in the firing of the postsynaptic neuron lose their power. In other words, classical Hebbian plasticity is caused by near-synchronized pre-synaptic and postsynaptic action potentials (APs) that result in an increase (LTP) or a decrease (LTD) of synaptic weights, respectively [2, 5, 7, 12].

We have highlighted the two main complementary representations of plasticity that are essential for the normal functioning of the nervous systems and which are different in their induction-dependency to pre-synaptic activity. Homosynaptic plasticity, which is a main guiding power for synaptic alterations that mediate associative learning, produces a favorable response to synaptic alterations, leading to unbalanced runaway dynamics. Furthermore, it does not cause the necessary degree of synaptic competition that is essential for various kinds of learning [13].

Another kind of plasticity is heterosynaptic plasticity. The impairment of molecular mechanisms of synaptic plasticity is very important and can be involved in neurological disorders such as Alzheimer’s disease [14]. Modeling research has demonstrated that heterosynaptic plasticity is required to drive and balance Hebbian plasticity. The exact relation between Hebbian and heterosynaptic plasticity is currently ambiguous in synaptic plasticity [11]. The excitatory–inhibitory balance that limits dendritic and somatic spiking is important for information processing in neural circuits [15]. Cortical inhibitory and excitatory inputs are initially incompatible but become balanced and co-tuned with experience by heterosynaptic plasticity [16]. Along with homosynaptic plasticity, the compensatory effect of heterosynaptic plasticity has since been detected between inputs synapsing onto the same neuron [16–19]. The compensatory role of heterosynaptic plasticity demonstrates that it can renormalize neuronal output, prevent runaway homosynaptic dynamics, and strengthen differences between synapses encoding distinct or opposing inputs [20].

In the present review, we will discuss the relationship between diverse plasticity systems and synaptic homeostasis, factors affecting the direction of plastic changes, and the role of heterosynaptic plasticity on the balance of synaptic weights.

## Multiple types of synaptic plasticity

Heterosynaptic and homosynaptic plasticity are balancing processes, and heterosynaptic plasticity might be associated with homosynaptic plasticity caused by distinctive pairing procedures [3]. Heterosynaptic plasticity counteracts runaway dynamics introduced by Hebbian-type rules and balances synaptic modifications. It provides learning systems with stability and augments synaptic competition [13].

The requirements for presynaptic activity for the induction of these types of plasticity are different. To induce homosynaptic plasticity at a synapse, pre-synaptic activation must exist. Furthermore, homosynaptic plasticity characterizes an input specificity or associativity that follows the rule of Hebbian-type learning [2]. The creation of associative homosynaptic plasticity needs associated activity between pre- and postsynaptic neurons [13].

Heterosynaptic plasticity happens at synapses influenced by distinctive ways of induction, such as tetanization [21, 22] or pairing protocols [23], even without the activation of presynaptic neuron [9]. An intense postsynaptic activity can induce heterosynaptic plasticity at synapses that were inactive in the induction time. Therefore, any synapse can be the target of heterosynaptic changes, whether active or not. Heterosynaptic plasticity can be triggered at unstimulated synapses using distinctive induction procedures or postsynaptic procedures, including afferent tetanization [21, 24], a pairing procedure [25], intracellular tetanization [22, 26], or bursts of spikes provoked by depolarizing pulses [22, 27]. Since most synapses delivered to a cell are not presynaptically stimulated in a distinctive induction, heterosynaptic plasticity impacts more synapses than homosynaptic plasticity [28]. Furthermore, evidence shows that the two may run individually or alongside each other at particular and important synapses of the mammalian brain. Both types can be formed by distinctive methods used to induce plasticity and work on the same timescales, but have varied computational properties and represent diverse effects on learning systems [13].

Heterosynaptic plasticity should operate on a timescale of seconds to minutes to inhibit runaway dynamics in neuronal networks and attain their stability, which is comparable to the timescale that homosynaptic plasticity is induced [13]. Changes in heterosynaptic plasticity at inactive synapses during plasticity induction yield stabilization of synaptic weights and intense synaptic competition in learning systems. This normalization implies that following a weight change at any synapse, all synaptic weights are normalized to maintain their total effect constant [29]. The total weight of synaptic inputs to a neuron could be preserved by local balancing of potentiation and depression [30]. This mechanism might not abolish the likelihood of saturating the potentiation or depression of a distinct synapse, but efficiently inhibits runaway activity.

While homosynaptic plasticity shows a greater specificity—and hence, a greater capacity for storing information—heterosynaptic plasticity results in a more long-lasting change in most systems. Meanwhile, homosynaptic plasticity has a specificity of connections and involves mainly learning and short-term memory, heterosynaptic plasticity regulates what information is stored as long-term memory [31]. In the hippocampal CA2/3a region, heterosynaptic plasticity and social memory were induced by enkephalin released from vasoactive intestinal peptide interneurons [32].

Another type of plasticity, spike-timing-dependent plasticity (STDP), in single spines exhibits a classical Hebbian STDP learning rule. It is bidirectional, in which presynaptic input inducing postsynaptic spikes creates timing-dependent LTP and postsynaptic spikes before presynaptic activation of single dendritic spines causes timing-dependent LTD [33].

## Functional effects of homosynaptic plasticity

Synaptic plasticity by Hebbian-type learning represents the creation and fine-tuning of neuronal connectivity and, therefore, exhibits a fundamental role in forming the hardware for forthcoming neuronal computations. Throughout life, Hebbian-type plasticity comprises processes including learning of specific and recurring relations between sensory stimuli and behaviorally relevant events, associations between sensory stimuli, learning of motor programs, and sequences of behaviors to adapt organisms to the changing environment. Finally, homosynaptic plasticity is thought to provide a cellular basis that underlies new learning and memory formation [13]. The connectivity assembly of cortical networks is extremely dynamic. This continuing cortical rewiring is assumed to serve vital functions for learning and memory. Synaptic assembly exerts a central role in the organization of memory in cortical circuits and counteracts memories from subsequent alterations. Rewiring of synaptic connections onto certain dendritic branches may hence prevent forgetting in neural networks [34].

Local homosynaptic activity drives coordinated modifications at inactive heterosynapses. For example, the induction of plasticity at the spines of glutamatergic neurons can change the synaptic power of unstimulated adjacent spines and hence induce heterosynaptic plasticity [35]. Furthermore, the excitatory plasticity at a distinct spine depresses the adjacent GABAergic inhibitory synapses, whereas more distant ones are potentiated [36].

Some forms of functional plasticity and learning-induced spine plasticity contribute to the storage of memory [37]. Spine plasticity is prominent until adolescence and then decreases in adulthood to very low levels during development. Spine dynamics contain the construction, loss, and stabilization of spines that are modified by neuronal activity and developmental age [38]. Furthermore, long-term imaging studies of the living brain have revealed that the cortical connectivity structure is dynamic, with dendritic spines being added and deleted on the time scale of hours to days [39]. Clustered loss of spines near to new spines during the acquisition of initial memory, enables the dendrites to generate multiple memories [40]. Asymmetric voltage reduction in dendrites has been shown to induce hierarchical heterosynaptic plasticity [41].

## The defects of homosynaptic plasticity and the compensation

Associative synaptic plasticity which underlies learning possesses two defects that need to be compensated by some mechanisms. Hebbian-type plasticity rules yield positive feedback and runaway dynamics of synaptic weights. The runaway of synaptic weights results in the overexcitability or silencing of neurons. Potentiation and depression make synapses stronger and weaker, respectively, and hence enhance and reduce the probability of their involvement in neuronal firing. Therefore, a positive feedback loop either potentiates the synaptic weights to the highest level or depresses them to the lowest [28]. Additional mechanisms, not limited to activated synapses, are required to counteract positive feedback made by Hebbian-type rules on synaptic weight alterations and to cause normal operation of learning systems. For example, potentiation of the synapses of the hippocampus [42] and amygdala [30], activated during afferent tetanization was along with the depression of adjacent synapses and vice versa. Potentiation and depression can balance each other so that net synaptic weight is preserved. Maximal intracellular Ca^2+^ ([Ca^2+^]_in_) rise in the stimulated synapses induces potentiation [43, 44], while smaller [Ca^2+^]_in_ rises less than plasticity threshold, in distant synapses induces depression [45].

Another deficiency of Hebbian-type plasticity is the presentation of only a weak degree of competition between synapses [46], limited to the synapses receiving discrete input patterns [47]. Strong synaptic competition needs the potentiation or depression of synapses toward extreme weights. Synaptic competition may yield the abolition of the “wrong” connections, such as the separation of inputs from two eyes during the development of the visual cortex (Wiesel and Hubel, 1963; Thompson et al., 1983) or the development of other sensory representations [4, 48] and many learning tasks that include discrimination [49]. Synapses might represent competition for resources that are available but limited, such as shared energy, molecules, or plasticity factors [46, 50]. Thus, stabilization mechanisms are necessary to provide intrinsic competition between synapses. The selectivity of cortical neuron responses to visual stimuli has been shown to result from the total number of synapses activated by different stimuli and both strong and weaker synapses are involved in it [51, 52].

## The direction of plastic changes: LTP or LTD plasticity

Heterosynaptic plasticity has been detected in various preparations and expressed in multiple forms. Heterosynaptic LTP was first discovered following the pairing of one input to a CA1 neuron in adjacent synapses and even synapses close by [53–56]. Therefore, LTP procedures make plasticity not only at the activated synapses but also at those not active during the induction; input specificity breaks down at short distances.

**Fig. 1:**
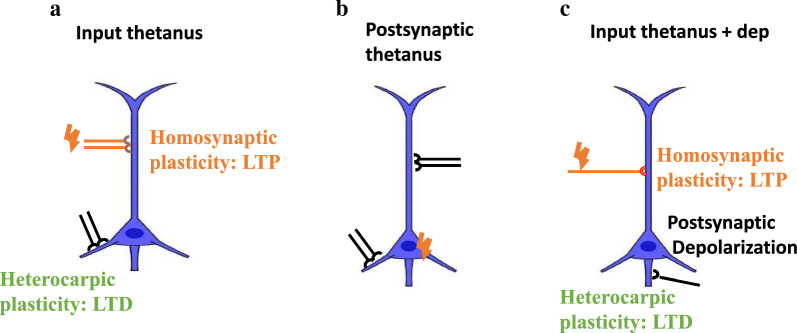
Multiple types of plasticity in neural synapses. **A** A schematic illustration of plasticity induction with its homo- and heterosynaptic pathways. Homosynaptic LTP (blue) induced by tetanus of input is associated with heterosynaptic LTD (black). **B** Heterosynaptic plasticity induction by postsynaptic tetanus, evoked by depolarizing pulses without pre-synaptic stimulation. **C** Mexican-hat profile of plasticity shows LTP induction by tetanus + postsynaptic depolarization at a set of synapses associated with a weaker heterosynaptic LTP at adjacent inputs and heterosynaptic LTD at inputs farther away (from Turrigiano et al., 1998)

Many types of heterosynaptic plasticity homeostatically control synaptic strength. For instance, homosynaptic LTP can cause heterosynaptic LTD at adjacent nonstimulated synapses [30, 57, 58]. Heterosynaptic LTD that is associated with LTP induction was first defined in the hippocampus (Fig. 1A) [59], where LTP was induced at the inputs from Schaffer collateral-commissural fibers to apical dendrites of CA1 pyramidal neurons. This LTP was associated with a heterosynaptic LTD at the inputs that reach the basal dendrites through commissural fibers, which were not stimulated during the induction. Also, the induction of LTP at the inputs to the basal dendrites was associated with heterosynaptic LTD of inputs to the apical dendrites (Fig. 1A). Heterosynaptic LTD is associated with the induction of homosynaptic LTP and exhibits the potential for both balancing plastic changes and supporting synaptic competition. Small subthreshold depolarization of the soma strongly boosts the propagation of hippocampal CA1 dendritic spikes and the induction of synaptic plasticity induced by the distal synaptic inputs [60].

Previous research has shown that beta-adrenergic receptor activation in the hippocampus induces heterosynaptic LTP through cyclic adenosine monophosphate (cAMP), protein kinase A and guanine nucleotide exchange protein activated by cAMP [61]. Cortical plasticity can be based on a heterosynaptic mechanism. For example, interhemispheric cortical LTP in the auditory cortex has been shown to need heterosynaptic activation of entorhinal projection [62]. In the hippocampus, Zn^2+^ released from mossy fiber to postsynaptic CA3 pyramidal cells facilitates the promotion of heterosynaptic potentiation of perforant path synaptic inputs through LTP of intrinsic excitability [63].

Heterosynaptic plasticity can be induced at unstimulated synapses using typical induction protocols such as afferent tetanization (Fig. 1A), purely postsynaptic protocols such as intracellular tetanization (bursts of spikes evoked by depolarizing pulses (Fig. 1B)), or a pairing procedure including input tetanization associated with postsynaptic depolarization (Fig. 1C). Long-term synaptic plasticity may be induced in both directions—potentiation and depression.

As Table 1 shows the direction of plastic changes depends on the following rules:

**Table 1:** The factors affecting the direction of plastic changes in synapses

	Factors induced long-term changes	LTP	LTD	References
1	Theta burst stimulation (TBS)	In synaptic inputs that received TBS	In other synaptic inputs without TBS	[23]
2	Frequency of tetanization	High > = 20 Hz	Low < = 3Hz	[64, 65]
3	Timing of the pre- to postsynaptic activity	10–20 ms before the postsynaptic cell	10–20 ms after the postsynaptic cell	[66, 67]
4	Intracellular Ca^2+^ concentration	Strong enhancement of [Ca^2+^]_in_	Moderate enhancement of [Ca^2+^]_in_	[27, 68, 69]
5	Pre-synaptic release probability	Lower initial release probability (high original PPR)	Higher release probability (low original PPR)	[22, 28, 71, 70, 72]
6	Sign of Hom-plasticity (LTP or LTD)	Het-LTP or Het-LTD at adjacent synapse	Het-LTD or Het-LTP at more distant inputs	[30, 54, 56]
7	Spatial distribution	LTP at more distant inputs	LTD at adjacent distances	[22]
8	History-dependency of synaptic inputs	Prior depression	Prior potentiation	[73, 74, 75, 76]
9	Firing of a third modulatory interneuron	Excitatory third neuron	Inhibitory third neuron	[77, 78, 79]

### Theta burst stimulation

**Fig. 2:**
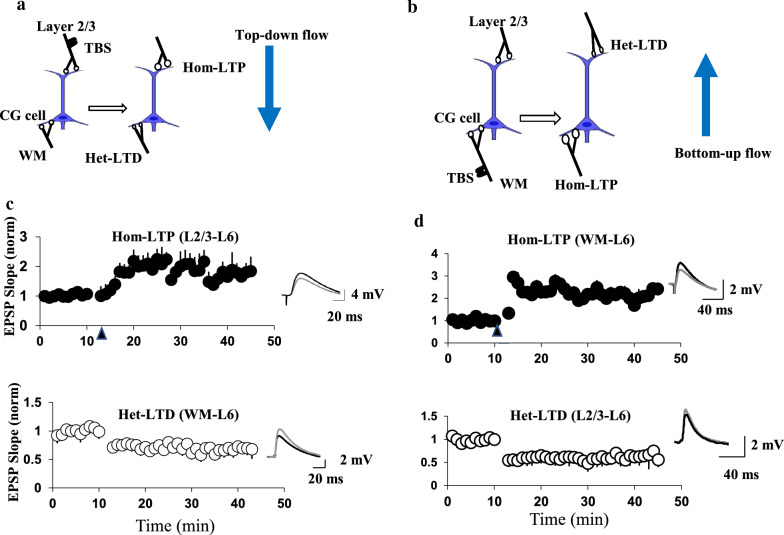
Persistent synaptic plasticity triggered by theta-burst stimulation (TBS). The schematic diagram of TBS application at synapses between layer 2/3 and corticogeniculate cells (CG) of layer 6 (**A**) and at synapses of white matter to CG cells in the visual cortex (**B**). **C** The graphs represent Hom-LTP of L2/3-evoked EPSPs (top) and het-LTD of WM-evoked EPSPs (bottom) triggered by TBS of the LII/III site. The means of EPSP slopes have been normalized to the control values 10–0 min before TBS for 14 cells as plotted against time. Circles and vertical bars indicate mean ± SEM. SEMs smaller than circles are not represented. The arrowheads below each plot exhibit the timing of TBS. **D** The graphs represent Hom-LTP of WM-evoked EPSPs initiated by TBS of the WM site and het-LTD of L2/3-evoked EPSPs (from Arami et al. [23])

In our earlier research, whole-cell recordings from corticogeniculate cells (CG) pyramidal neurons in layer VI of visual cortical slices were achieved under infrared differential interference contrast optics. Two stimulating electrodes were positioned in layer II/III adjacent to the margin of layer IV and at the position in the white matter (WM) to stimulate two main afferents to layer VI neurons. Theta burst stimulation (TBS) of layer II/III induced homosynaptic LTP (hom-LTP) and heterocarpic LTD (het-LTD) at layer II/III to layer IV and WM to layer IV synapses, respectively. In TBS to WM, this mode is switched to the opposite (Fig. 2) [23]. In another study, when the strong TBS preceded the weak TBS, heterosynaptic plasticity can be induced through calcium-permeable AMPA receptors [80].

### Frequency of tetanization

**Fig. 3:**
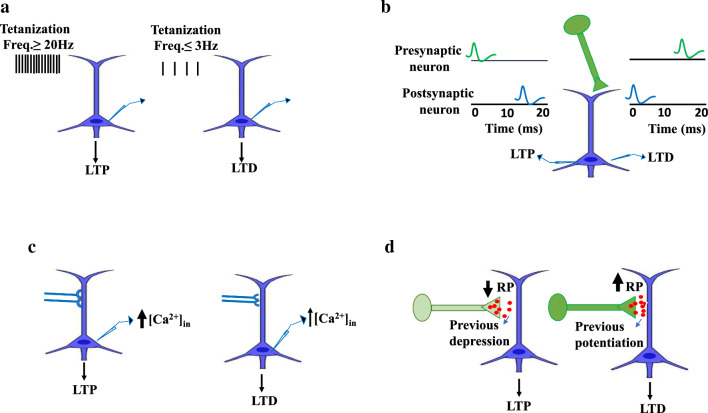
Determinants of the direction of synaptic plasticity. **A** The high and low frequency of tetanization determines the kind of plasticity. **B** Activation of the synapses 10–20 ms before or after the firing of the postsynaptic cell leads to the induction of LTP or LTD, respectively. **C** High and minor augmentation of [Ca^2+^]_in_ could induce LTP and LTD, respectively. **D** Weak inputs (light green input) with low initial release probability (RP) were classically potentiated, while strong inputs (dark green input) with a high initial release probability were typically depressed or did not alter (from Turrigiano et al., 1998)

In afferent tetanization, frequency determines the direction of plasticity. High-frequency tetanization (20 Hz and above) results in potentiation, but low frequency (3 Hz and below) results in depression (Fig. 3A) [64, 65].

### Timing of the pre- to postsynaptic activity

It was revealed that activating the synapses in the hippocampus and the neocortex 10–20 ms before or after the postsynaptic cell fires leads to the induction of LTP or LTD, respectively (Fig. 3B) [66, 67].

### Intracellular Ca^2+^ concentration

Heterosynaptic plasticity can also be induced by distance-independent mechanism(s) through the increase of intracellular Ca^2+^ concentration triggered by the photolytic release of caged Ca^2+^ [68] or intracellular tetanization and without any pre-synaptic stimulation [27, 69]. In the hippocampal CA1, the pairing of one input to a pyramidal neuron resulted in the potentiation of that stimulated synapse, nearby synapses, and even close neurons [11]. This kind of heterosynaptic plasticity is not involved in the pre-synaptic stimulation of the synapse and is purely induced by postsynaptic protocols. A rise in intracellular calcium concentration may trigger heterosynaptic plasticity. Evidence shows that the chelation of intracellular calcium with EGTA can block long-standing heterosynaptic plasticity [22, 26, 81]. In the hippocampus and neocortex, rises in intracellular calcium concentration or postsynaptic spiking can induce plasticity [11].

The direction of plastic changes underlies intracellular calcium dynamics; large and small augmentation of [Ca^2+^]_in_ could induce LTP and LTD, respectively (Fig. 3C) [82–84]. Calcium dynamics are extremely predictive of the change in synaptic weight. Calcium dynamics are affected by local synaptic activity and depolarization [85]. Voltage-dependent calcium channels that are activated by back-propagating action potentials lead to the enhancement of [Ca^2+^]_in_, even in non-active dendrites [13] that can support backpropagation and the rate of firing [86, 87]. Sites with a strong or moderate enhancement of [Ca^2+^]_in_ will produce heterosynaptic LTP or LTD, respectively. Since strong local stimulation that leads to [Ca^2+^]_in_ increase is not limited to the stimulated synapses, it can activate positional heterosynaptic plasticity at adjacent sites. Certainly, the profile of [Ca^2+^]_in_ increase near the induction location can determine the potentiation and depression induced in the activated dendrite [30]. The release of calcium from internal supplies may simplify the induction of this heterosynaptic plasticity [30, 88, 89].

### Pre-synaptic release probability

In intracellular tetanization, bi-directional fluctuations of synaptic transmission are associated with the features of pre-synaptic release mechanisms like paired-pulse facilitation (PPF) ratio. Following intracellular tetanization, synapses with a high PPF ratio, which has a low release chance, were mostly potentiated. The synapses with low PPF ratio and high release probability were either mostly depressed or simply did not demonstrate any change [70]. The track of the plastic changes of a synaptic input was linked to the original paired-pulse ratio, which is inversely related to release probability [22, 28, 71]. Inputs with lower initial release probability were classically potentiated. Inputs with a higher release probability were classically declined or did not alter (Fig. 3D) [82–84].

### Sign of homosynaptic plasticity

**Fig. 4:**
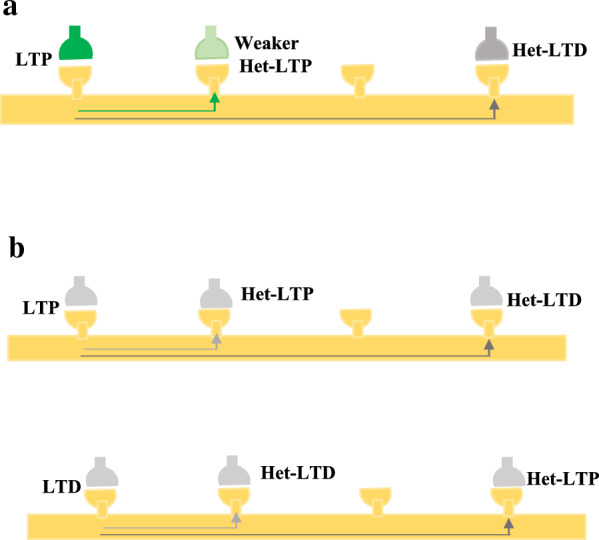
The effect of spatial distribution and sign of homosynaptic plasticity on the direction of synaptic plasticity. **A** Inducing LTP at a fraction of synapses was associated with a weaker heterosynaptic LTP at adjacent distances and heterosynaptic LTD at more distant inputs. **B** Heterosynaptic plasticity with the same homosynaptic plasticity is induced at short distances, while those of opposite signs have been induced further away from the focus of activation

Heterosynaptic plasticity with the same homosynaptic plasticity is induced at short distances [30, 54, 56], while those of opposite signs have been induced further away from the focus of activation [30] (Fig. 4, B).

The sign of synaptic plasticity is structured by the distribution of the backpropagating action potential to the synapse. This produces a gradient between LTP and LTD as their distance from the soma rises. Cooperative synaptic input or dendritic depolarization at distal synapses can switch plasticity between LTD and LTP by enhancing the backpropagation of action potentials. This activity-dependent shift makes an associative learning mechanism that functions across various neocortical layers [90].

### Spatial distribution

One of the factors affecting the direction of plasticity is being far from the site of activation when plasticity is induced [30, 42]. The mapping of neuronal connectivity on multiple spatial scales has been developed [91–93]. Potentiation and depression can occur at synapses with comparable distances and, hence, similar calcium rises [22]. Still, the tendency of a synapse for plasticity affects whether it experiences LTP or LTD [13]. LTP and LTD represent a bi-phasic spatial distribution in the hippocampus and amygdala, which have a regular organization of inputs [30, 42]. Inducing LTP at a fraction of synapses was associated with a weaker heterosynaptic LTP at inputs close by and heterosynaptic LTD at those that were farther away. A regular shape of heterosynaptic alterations was detected at the induction site of LTD: weaker LTD at adjacent distances and LTP at more distant inputs [30] (Fig. 4A).

Evidence retrieved from the balanced profiles of plastic changes between potentiation and depression shows that the net alterations of the total synaptic input at activated synapses are neither affected by LTP nor LTD induction. Therefore, synaptic weights and synaptic competition can be normalized by the powerful local mechanism of normalization that this type of heterosynaptic plasticity provides [11]. Heterosynaptic plasticity depends on how far the stimulated synapses are during induction. Shorter distances experience same-sign plasticity while farther distances cause opposite-sign plasticity [30, 42], which may provide lateral inhibition in plasticity space, highlighting the effect of plastic change at a local population of synapses and distinguish them from other synapses [56].

LTP can be communicated between synapses on adjacent neurons through a diffusible messenger. This spread of potentiation provides a mechanism for the cooperative strengthening of proximal synapses [56]. Synapses are potentiated regardless of their activation history if they are adjacent to a spot of potentiation, while synapses farther away display no potentiation [54]. It has been shown that the induction of LTP at an individual glutamatergic spine depresses the nearby inhibitory synapses (within 3 μm), whereas more distant ones are potentiated [36]. In macaque V1, the stimulus feature affects the spatial distribution of synaptic inputs on dendrites. Synaptic inputs on dendrites are also functionally distributed in multidimensional feature space. These items provide a likely substrate of local feature integration on dendritic branches [94].

### History-dependency of synaptic inputs

**Fig. 5:**
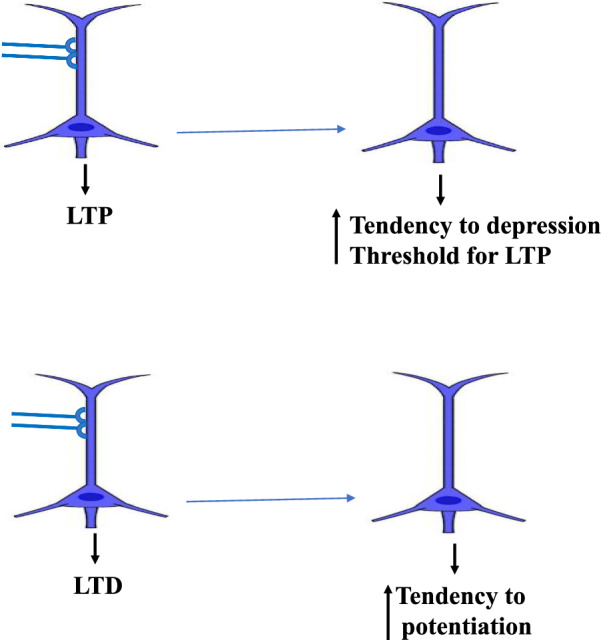
History-dependency of synaptic plasticity. Weak synaptic inputs with low release probability which have undergone depression in the past have a stronger disposition for potentiation. Strong synapses with a high release probability, which have been potentiated recently, show a higher tendency for depression

The weight-dependence of heterosynaptic plasticity might show the history-dependency of synaptic inputs to undertake potentiation or depression [3]. Prior potentiation results in a higher predisposition for depression or depotentiation [73] and may augment the threshold for LTP induction [74]. Weak synaptic inputs with low release probability which have undergone depression in the past have a stronger disposition for potentiation [75, 76]. In other words, inputs with a high original PPF ratio exhibit more pronounced potentiation [72]. Strong synapses with a high release probability, which have been potentiated recently, show a higher tendency for depression [75, 76]. Volgush et al. pointed out that the PPF ratio rises in LTD induction [72]. Therefore, the potentiation or depression of heterosynaptic alteration is related to the previous experience of the synapse which has undergone depression or potentiation in the past (Fig. 5).

### Firing of a third modulatory interneuron

**Fig. 6:**
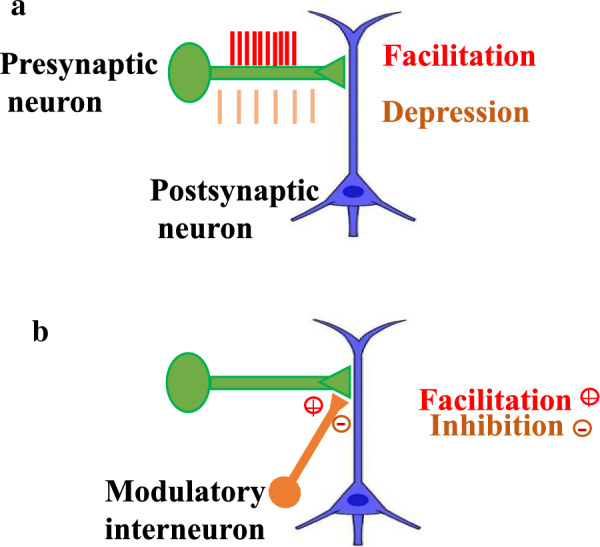
Homosynaptic and heterosynaptic changes in long-lasting plasticity. **A** All the required events for synaptic strengthening or weakening arise from the same synapse in homosynaptic plasticity. These alterations may cause an enhancement (homosynaptic facilitation) or a reduction in synaptic strength (homosynaptic depression). **B** The firing of a third neuron, a modulatory interneuron whose terminals end on the synapse, can adjust the strength of the specific synapse. These alterations may cause a rise (heterosynaptic, modulatory facilitation) or a decline (heterosynaptic inhibition) in synaptic strength (from Bailey et al. [77])

According to Hebb, in homosynaptic plasticity, all the required synaptic strengthening or weakening events arise from the same synapse. These alterations may cause an enhancement (homosynaptic facilitation) or a reduction in the synaptic strength (homosynaptic depression) (Fig. 6A) [77]. Kandel and Tauc proposed that the strength of a synapse would increase or decrease by the firing of a third modulatory interneuron, without the activity of either the pre- or postsynaptic neurons (Fig. 6B). They pointed out that this heterosynaptic modulation could have one of two types: non-associative or associative [77]. The non-associative system is heterosynaptic, while in the associative form, activity-dependent heterosynaptic modulation associates the homosynaptic and heterosynaptic mechanisms. If the firing of the modulatory input is correlated in time with the firing of the pre-synaptic cell, its strengthening effect will increase [78, 79]. Therefore, heterosynaptic plasticity that is caused by strong postsynaptic activity may break the runaway dynamics of synaptic weights. Heterosynaptic plasticity that does not require pre-synaptic activity at the synapse for induction is triggered by the augmentation of intracellular calcium and activated on the same timescale as homosynaptic plasticity. Furthermore, heterosynaptic alterations can be induced by the same methods that are utilized to induce homosynaptic plasticity [11].

## Trigger for heterosynaptic plasticity

It has been shown that the fundamental factor affecting the induction of heterosynaptic plasticity is the firing rate of the postsynaptic neuron. The postsynaptic activation is affected by the strength of the stimulus and, hence, by the number of synchronized stimulated synapses [95]. Therefore, a cluster of neuronal inputs on a postsynaptic neuron would induce heterosynaptic plasticity if they also enhance postsynaptic activity. Moreover, the number of synchronized synapses depends on the activity-dependent structural alterations [96, 97].

The induction of heterosynaptic and homosynaptic plasticity correlates with the postsynaptic calcium concentration [30, 45]. Increases of [Ca^2+^]_in_ during Hebbian-type and heterosynaptic plasticity induction [22, 45, 98] are not restricted to the activated synapses only, and can be evoked by bursts of backpropagating APs even without synaptic activation [43, 87, 90, 99, 100]. Calcium chelation inhibits the induction of heterosynaptic plasticity [22]. Therefore, a calcium-based plasticity form that induces homosynaptic (spike-timing-dependent) plasticity [101, 102] may induce heterosynaptic variations.

## Intracellular tetanization-induced heterosynaptic plasticity

While long-term variations in synaptic transmission in rat visual cortex were induced either by pairing the excitatory postsynaptic potentials with postsynaptic depolarization or by intracellular tetanization without synaptic stimulation, successive application of two protocols impaired the maintenance of long-term potentiation [103].

Bursts of spikes induced by short depolarizing pulses through the recording electrode, intracellular tetanization, induce heterosynaptic plasticity [3, 22]. In intracellular tetanization, each neuron collects many synaptic inputs, but only a fraction of them are required to be activated to induce spikes. Repetitive activation of a portion of inputs and the resultant repetitive firing of the postsynaptic cell can cause heterosynaptic plasticity [11]. A postsynaptic activation that does not include pre-synaptic activity is similar to intracellular tetanization. Since none of the synaptic inputs were evoked through intracellular tetanization, any alteration in synaptic transmission after intracellular tetanization can be thought of as heterosynaptic plasticity. Postsynaptic activity by intracellular tetanization is compatible with the activity patterns that have been detected similar to postsynaptic activity in distinctive plasticity-induction procedures [11]. Amplitudes of synaptic responses will enhance, diminish, or remain unchanged after intracellular tetanization. Furthermore, intracellular tetanization synchronously can induce LTP and LTD onto the same cell in two independent inputs [104].

## Other mechanisms of heterosynaptic plasticity

Intracellular tetanization-induced plasticity comprises pre-synaptic components, indicating retrograde signaling [22, 27, 72]. One retrograde messenger is nitric oxide (NO). Diffusion of NO that is formed in stimulated synapses mediates local heterosynaptic plasticity of both excitatory and inhibitory transmission [56, 81, 105, 106]. The plasticity triggered via intracellular tetanization depends on the original paired-pulse ratio that is inhibited by blocking NO-synthase [22, 27]. NO signaling required strong intracellular tetanization, because plasticity induced by weaker postsynaptic activity did not correlate with the initial paired-pulse ratio [27]. Astrocytes not only affect the activity of single synapses but also are the main essentials in the experience-dependent wiring of brain circuits. Astrocytes regulate experience-dependent plasticity in the mouse visual cortex during the critical period [107].

Heterosynaptic LTD can be induced by the activity-dependent release of adenosine three phosphate (ATP) and adenosine from neurons [108] and astrocytes [28, 109, 110] that are inhibited by the adenosine receptor antagonist [108, 109], and diminished by A1 receptor inactivation [109]. Astrocytes have been shown to control the polarity of NMDA receptors and adenosine-dependent presynaptic plasticity [111]. In another study, adenosine reinforced weight dependence of heterosynaptic plasticity and inhibition of adenosine A1 receptors impaired it [112].

It has been found that heterosynaptic potentiation depends on inhibition mediated by gamma-aminobutyric acid (GABA)_A_ receptors. Hyperpolarization induced by the GABA_A_ receptor may be essential to deactivate the T-type calcium channels of the apical tuft. This prospect is consistent with evidence showing that R- or T-type Ca^2+^ channels are needed for heterosynaptic potentiation [26]. Short- and long-term plasticity at both the pre-and postsynaptic levels can be formed in GABAergic synapses [113, 114]. Weight-dependent heterosynaptic plasticity may be an ideal candidate mechanism to accomplish homeostatic regulation of synaptic weight at excitatory synapses to inhibitory neurons [115].

## Diverse functions of different kinds of heterosynaptic plasticity

Heterosynaptic plasticity is a central mechanism for some functional characteristics of neuronal circuits [46]. It has been demonstrated that homosynaptic potentiation may cause a type of heterosynaptic potentiation [54, 116] that influences a much broader spatial scale and has directional and input specificity. Therefore, inducing one group of inputs can affect other anatomically and functionally different sets. Synapses onto distal apical tuft are assumed to modulate time-locked proximal inputs [117–119]. Apical tuft synapses might control the activity of neurons through heterosynaptic potentiation of proximal synapses [26]. In macaque V1, apical dendrite inputs have greater receptive fields and hence show a main role in integrating feedback in information processing [94]. Furthermore, extensive response latencies of apical dendrites demonstrate that they receive feedback inputs, while basal dendrites receive feedforward inputs within the visual hierarchy [120, 121].

Several kinds of heterosynaptic plasticity homeostatically control synaptic strength; for instance, homosynaptic LTP can cause heterosynaptic LTD at adjacent nonstimulated synapses [30, 57, 58]. One distinctive feature of this kind of heterosynaptic plasticity is the short spatial spread of its effects, which is attributable to the short-range diffusion of underlying signaling molecules like endocannabinoids or internal Ca^2+^ [26].

Homosynaptic LTP and heterosynaptic LTD may modify the synaptic transmission effectiveness through diverse mechanisms. For example, in our earlier research, we showed that the CG cells of layer VI in the visual cortex receive top-down synaptic inputs from upper layers and bottom-up sensory inputs. We concluded that hom-LTP and het-LTD can increase and decrease the peripheral impact on the gain control function of layer VI neurons, respectively. Therefore, heterosynaptic plasticity may switch the principal stream of information flow from one-layer VI visual cortex neuron to the other. Another possible functional significance of reciprocal het-LTD may be the stability of synaptic function. As pointed out in previous studies, the one-way action of synaptic plasticity, such as hom-LTP, would saturate synaptic transmission efficacy. Hence, het-LTD might be needed to stabilize the effectiveness of synaptic transmission and prevent synapses that receive extremely repetitive inputs from being saturated [23].

The principal inhibitory neurons in the neocortex are fast-spiking (FS) and non-fast spiking (non-FS), which have different roles and properties [122]. Recently, weight-dependent heterosynaptic plasticity has been considered a new form of plasticity in excitatory synapses on both FS and non-FS inhibitory neurons [28, 123]. This kind of plasticity is an extensive phenomenon that could play a role in inhibiting runaway dynamics at excitatory synapses and lead to potentiation or depression [11, 123]. Interestingly, heterosynaptic plasticity shows different net impacts in FS and non-FS cells [123]. Heterosynaptic alterations in FS neurons preserve overall excitation/inhibition balance [124–126] while permitting local activity reorganization and synchronization [123]. In non-FS neurons, heterosynaptic plasticity may inhibit the removal of low-probability synapses via Hebbian-type plasticity and, hence, maintain inhibitory neurons stimulated by these synapses. The evidence shows that GABA and NO are contributed to retrograde signaling related to the induction of heterosynaptic plasticity in pyramidal and inhibitory neurons [12, 127]. Our previous study discovered that tetanic activation of pre-synaptic FS-GABA neurons in layer II/III of the mouse visual cortex induced LTP, whereas that of pre-synaptic non-FS spiking GABA neurons could not produce LTP. We proposed that the long-standing plasticity of inhibitory synapses on FS GABA neurons is pathway-specific. Furthermore, we concluded that P/Q-type channels might contribute to LTP production in inhibitory synapses among FS-GABA neurons [128].

Previous studies showed that heterosynaptic plasticity substantially develops synaptic dynamics and neuronal functionality and, hence, complex neural circuits [55]. Moreover, heterosynaptic plasticity inhibits the homosynaptic-induced deviations of the synaptic dynamics and stabilizes neural circuits [28].

Alterations of the power of neuronal contacts are widely considered to be the mechanism for encoding and storing memory traces in the central nervous system. During memory formation, activity-dependent synaptic plasticity is induced at appropriate synapses. Much data have shown that synaptic plasticity is required for learning and memory, but few demonstrate the concept of sufficiency [129]. It has been demonstrated that synaptic plasticity mediates learning and is essential for neuroscience. Changed heterosynaptic plasticity weakens visual discrimination learning in adenosine receptor knock-out mice [130].

Heterosynaptic LTP is different from Hebbian LTP. Whereas LTP develops rapidly without any alteration in N-Methyl-D-aspartate (NMDA) receptor-mediated currents, heterosynaptic plasticity is expressed slowly with changes in NMDA receptor subunits. Therefore, LTP and heterosynaptic plasticity are appropriate for encoding memories. CA3 firing rates continue within minutes in a new environment, while CA1 firing lasts over hours to days [131–134]. It has been shown that dendritic NMDA spikes are essential for timing-dependent associative LTP in CA3 pyramidal cells [135]. Furthermore, NMDA receptor-dependent multidendrite Ca^2+^ spikes are required for hippocampal burst firing [136]. In thin dendrites of CA1 pyramidal neurons, input patterns evoking dendritic spikes can reinforce nonsynchronous synapses by local heterosynaptic plasticity [137].

## Conclusion

Multiple mechanisms mediate heterosynaptic plasticity. Heterosynaptic plasticity that is triggered by intracellular tetanization demonstrates features that are well appropriate for normalizing synaptic weights: (a) it dampens strong synapses and potentiates the weak ones, hence, ending the runaway dynamics of synaptic weights, (b) it can be evoked at non-active synapses, and (c) it activates the same timescale as homosynaptic plasticity. The direction of heterosynaptic plasticity can be determined by factors such as frequency of tetanization, the timing of the pre- to postsynaptic activity, distance, pre-synaptic release probability, history of synaptic inputs, the sign of homosynaptic plasticity, and firing of a third modulatory interneuron. In addition, cell-wide signals can cause heterosynaptic changes, including [Ca^2+^]_in_ rises induced by backpropagating action potentials. Even though the associative activation of pre-synaptic sites is not required in heterosynaptic plasticity, inducing is nevertheless almost surely determined by these signals.

## Data Availability

Not applicable.
